# Supplementary material for: Myc-Interacting Zinc Finger Protein 1 (Miz-1) Is Essential to Maintain Homeostasis and Immunocompetence of the B Cell Lineage
Source: Biology (Basel). 2022 Mar 24;11(4):504. doi: 10.3390/biology11040504 (PMC9027237; doi:10.3390/biology11040504)
Supplement: Supplementary file 1 [file biology-11-00504-s001.zip › Supplementary_information.pdf]

Supplementary information

Supplementary materials and methods

Immunofluorescence of Peyer's patches.

For immunohistochemical analysis, tissue specimens were collected at the time of autopsy and processed for cryo-conservation. Peyer's Patches were equilibrated 5 minutes on ice in O.C.T. medium (Sakura Finetek Europe B.V), transferred to fresh O.C.T medium and snap-frozen in liquid nitrogen. For immunofluorescence 8 µm sections were mounted on charges microscopy slides and air-dried for 10 min at RT. Tissue was fixed with ice-cold acetone for 10 min at -20°C and washed with TBS-T. Tissue specimens were blocked with 1% BSA and incubated with fluorescence-coupled antibodies diluted 1:200 over night at 4°C in a humid chamber. Nuclei were counterstained with 1 µg/ml DAPI (7min, RT) and tissue staining was conserved with ProLong™ Diamond Antifade Mountant (invitrogen) and air-dried for 24h at RT prior to analysis. Microscopic pictures were acquired with Zeiss LSM 880 with Airyscan. Pictures were adjusted with ZEN Microscopy and image software.

Immunization of mice.

Immunization was performed as defined in the animal test request (#01-072/14) with 8-14 week old male and female mice and *i.p.* injection of 50 µg NP(16)-CGG (Biosearch Technologies) and Imject® Alum (Thermo Scientific) as adjuvant. A second booster immunization was performed 30 days post-immunization. Animals were sacrificed by CO_2 asphyxiation 7 days post booster immunization and analyzed by flow cytometry.

Table S1. Primer sequences.

Gene	Forward Primer (5'-3')	Reverse Primer (5'-3')
bactin	CTAAGGCCAACCGTGAAAAG	ACCAGAGGCATACAGGGACA
Zbtb17	AGTGCAGCAAGGCTTTCTCGGA	AGGCTGATGAGCCTGTAGCTCT
E2A	GTGGATGATGAACCAGTCTCAG	ACAGGTAGCGGGAACATCA
Ebf1	CCAACTCACCCTATGCCATT	GGGGAGGCTTGTAGATGAGG
Pax5	GACTCCTCGGACCATCAGG	GGCCGTCCATTCACAAAA
Id2	TCACCAGAGACCTGGACAGAAC	TGCTATCATTCGACATAAGCTCAG
Tnfa	GGTGCCTATGTCTCAGCCTCTT	GCCATAGAACTGATGAGAGGGAG
Myc	TTTGTCTATTTGGGGACAGTGTT	CATCGTCGTGGCTGTCTG

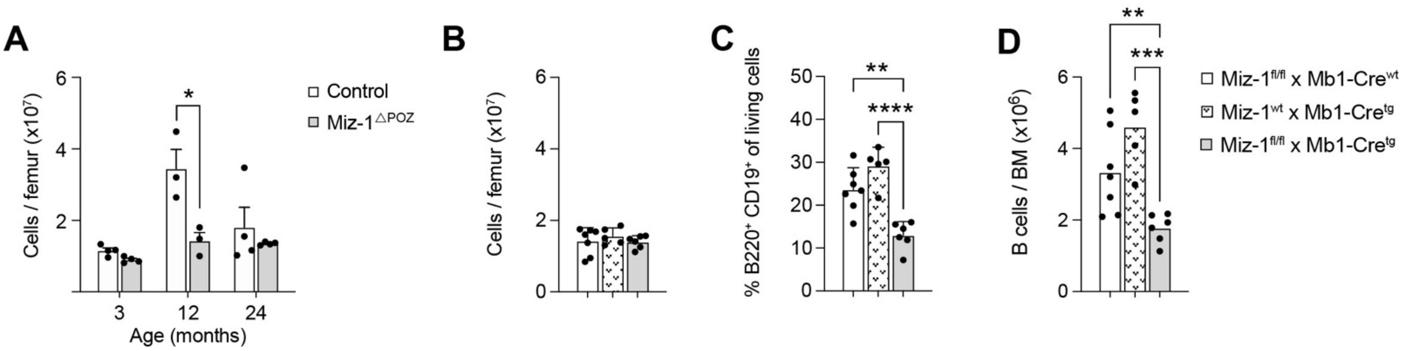

Figure S1. Cell counts of bone marrow and late B cells. (A) Absolute numbers of bone marrow cells of control and Miz-$1^{\Delta POZ}$ mice at indicated ages. **(B)** Absolute number of bone marrow cells of 3 months old Miz-$1^{fl/fl}$ x Mb1-Cre^{wt} (Control), Miz-1^{wt} x Mb1-Cre^{tg} and Miz-$1^{fl/fl}$ mice x Mb1-Cre^{tg} (Miz-$1^{\Delta POZ}$). **(C)** Frequency of B cells ($B220^{+}CD19^{+}$) from bone marrow of Miz-$1^{fl/fl}$ (Control), Miz-1^{wt} x Mb1-Cre and Mb1-Cre x Miz-$1^{fl/fl}$ mice (Miz-$1^{\Delta POZ}$) mice at 3 months. **(D)** Quantification of absolute numbers of B cell numbers ($B220^{+}CD19^{+}$) from (C) in the bone marrow. Each point represents one mouse. Data are expressed as mean + SD (n≥3). Student's unpaired t-test (A) and one-way ANOVA (C,D): *p<0.05, **p<0.01, ***p<0.001, ****p<0.0001.

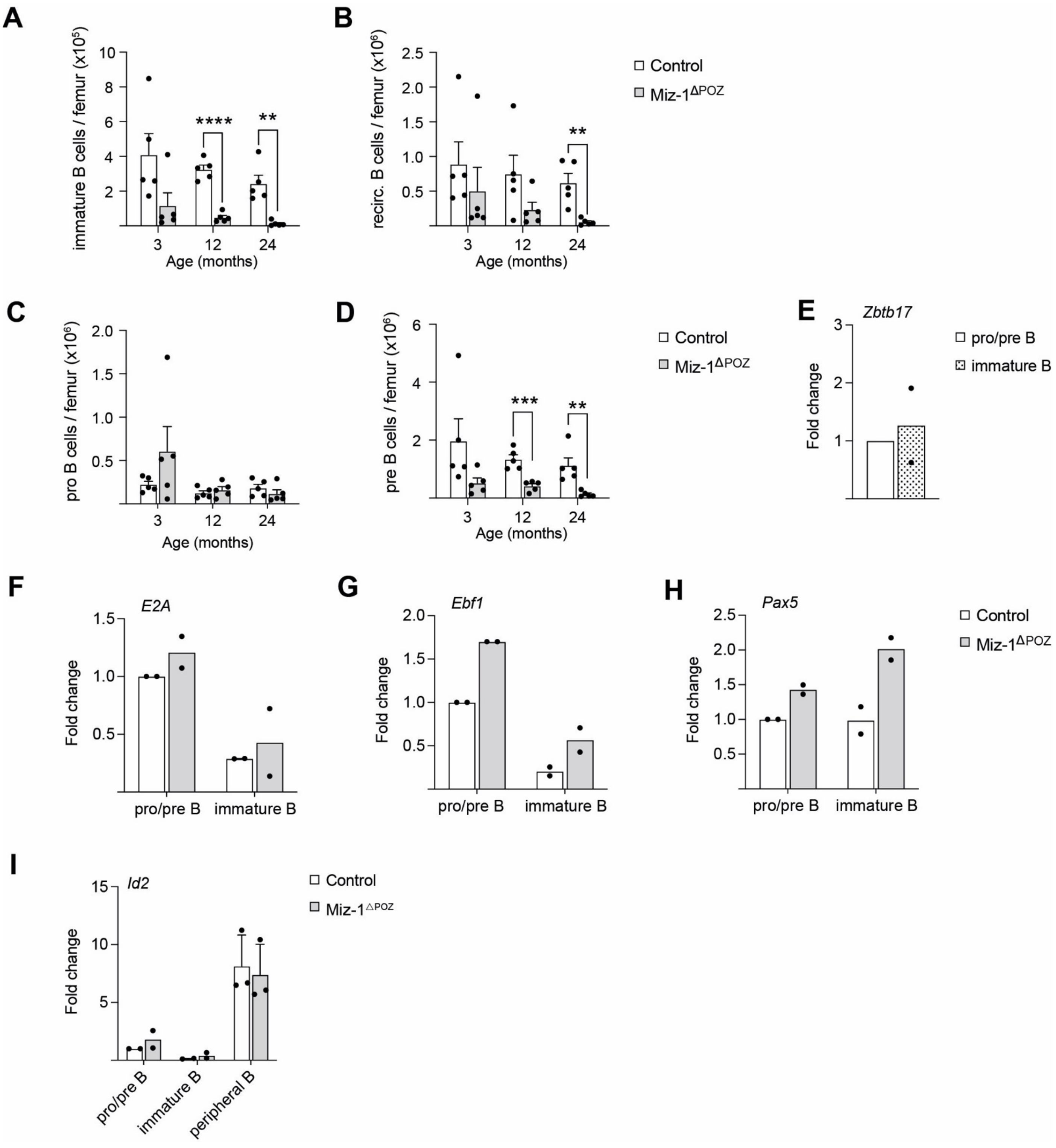

Figure S2. Quantification of different B cell maturation stages. (A) Quantification of absolute numbers of immature B cells ($B220^+CD19^+CD93^+CD43^-IgM^+$) from the bone marrow. **(B)** Quantification of absolute numbers of recirculating B cells ($B220^+CD19^+CD93^-CD43^-IgM^+$) from the bone marrow. One point represents one mouse. **(C)** Quantification of absolute numbers of pro B cells ($B220^+CD19^+CD93^+CD43^+IgM^-$) from the bone marrow. **(D)** Quantification of absolute numbers of pre B cells ($B220^+CD19^+CD93^+CD43^-IgM^+$) from the bone marrow. **(E)** Expression of *Zbtb17* (Miz-1) in sorted B cell precursors B from old control animals determined by qRT-PCR. Expression was normalized to pro/pre B cells. **(F)** Expression of *E2A* in B cell precursors from aged (12 months) control and Miz-1$^{\Delta POZ}$ animals determined by qRT-PCR. Expression was normalized to pr/pre B cells. **(G)** Expression of *Ebf1* in B cell precursors from aged (12 months) of control and Miz-1$^{\Delta POZ}$ animals determined by qRT-PCR. Expression was normalized to pro/pre B cells. **(H)** Expression of *Pax5* in B cell precursors from aged (12 months) of control and Miz-1$^{\Delta POZ}$ animals determined by qRT-PCR. Expression was normalized to pro/pre B cells. **(I)** Expression of *Id2* in B cell precursors and peripheral B cells B from aged (12 months) of control and Miz-1$^{\Delta POZ}$ animals determined by qRT-PCR. Expression was normalized to pro/pre B cells.
Each point represents one mouse. Data are expressed as mean + SD (n≥2). Student's unpaired t-test: $^{**}p<0.01$, $^{***}p<0.001$.

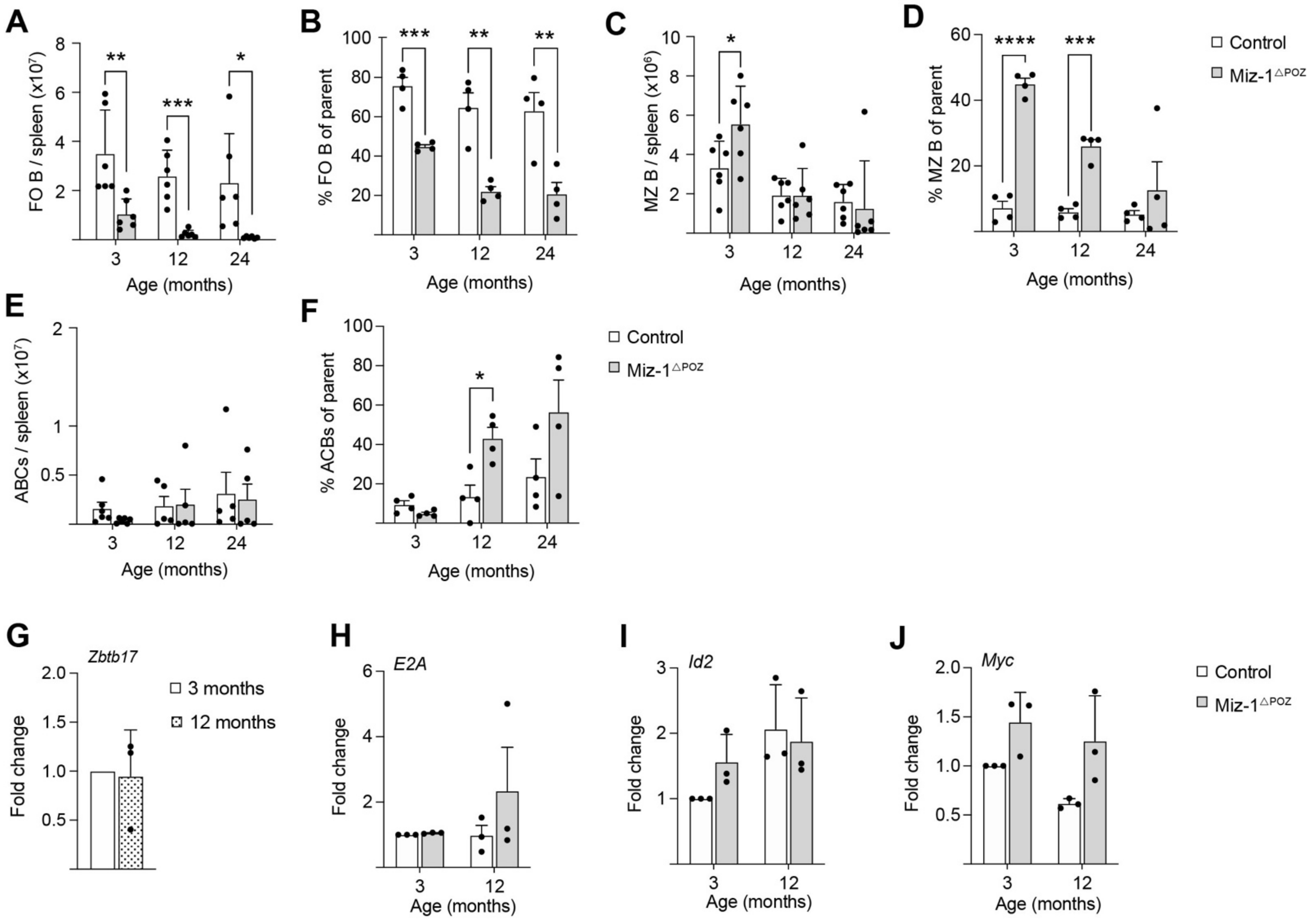

Figure S3. Analysis of splenic B cell population. (A) Quantification of splenic FO B cells ($CD19^{+}CD93^{-}CD21^{med}CD23^{+}$) in the spleen from control and Miz-$1^{\Delta POZ}$ mice. **(B)** Frequency of FO B cells relative to $CD19^{+}CD93^{-}CD43^{-}$ cells. **(C)** Quantification of splenic marginal zone B cells ($CD19^{+}CD93^{-}CD21^{+}CD23^{-}$) in the spleen from control and Miz-$1^{\Delta POZ}$ mice. **(D)** Frequency of MZ B cells relative to $CD19^{+}CD93^{-}CD43^{-}$ cells. **(E)** Quantification of age-associated B cells ($CD19^{+}CD93^{-}CD21^{-}CD23^{-}$) in the spleen from control and Miz-$1^{\Delta POZ}$ mice. **(F)** Frequency of ABCs relative to $CD19^{+}CD93^{-}CD43^{-}$ cells. Each point represents one mouse. Data are expressed as mean + SD ($n \geq 4$). Student's unpaired t-test: *p<0.05, **p<0.01, ***p<0.001.

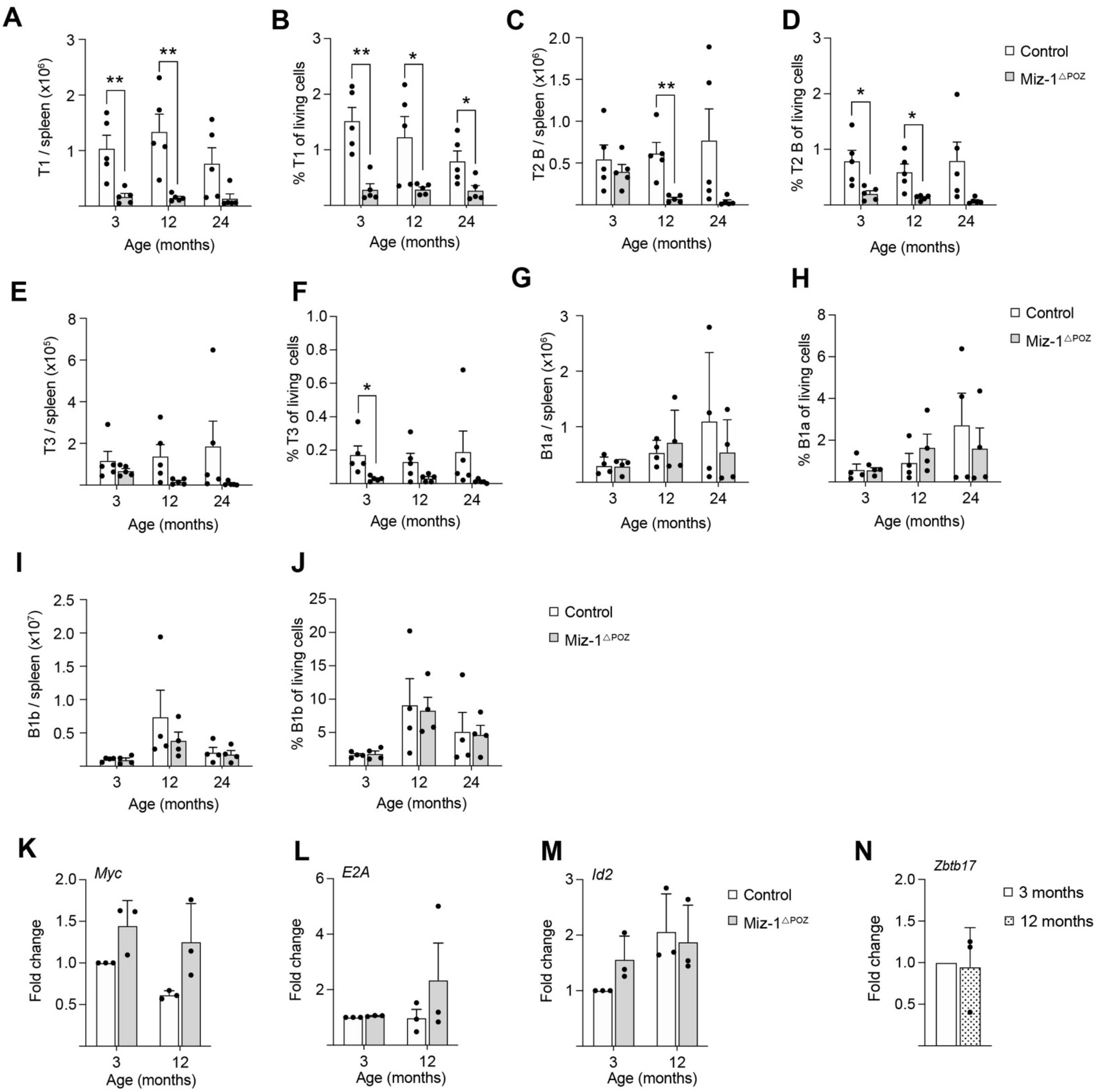

Figure S4. Splenic B cells and gene expression. (A, C, E, G, I) Quantification of T1 ($CD19^{+}CD93^{+}IgM^{high}CD23^{-}$), T2 ($CD19^{+}CD93^{+}IgM^{high}CD23^{+}$), T3 ($CD19^{+}CD93^{+}IgM^{low}CD23^{+}$), B1a ($CD19^{+}B220^{low/med}CD43^{+}CD5^{+}$) and B1b ($CD19^{+}B220^{low/med}CD43^{+}CD5^{-}$) B cells in the spleen from control and Miz-$1^{\Delta POZ}$ mice. **(B, D, F, H, J)** Frequency of T1, T2, T3, B1a and B1b B cells in the spleen from control and Miz-$1^{\Delta POZ}$ mice. **(K-M)** Expression of *Myc*, *E2A* and *Id2* in splenic B cells from young (3 months) and aged (12 months) of control and Miz-$1^{\Delta POZ}$ animals determined by qRT-PCR. Expression was normalized to young B cells. **(N)** Expression of *Zbtb17* (Miz-1) in peripheral B cell from 12 months old animals determined by qRT-PCR. Expression was normalized expression of control animals.
Each point represents one mouse. Data are expressed as mean + SD (n≥3). Student's unpaired *t*-test: *p<0.05, **p<0.01, ***p<0.001.

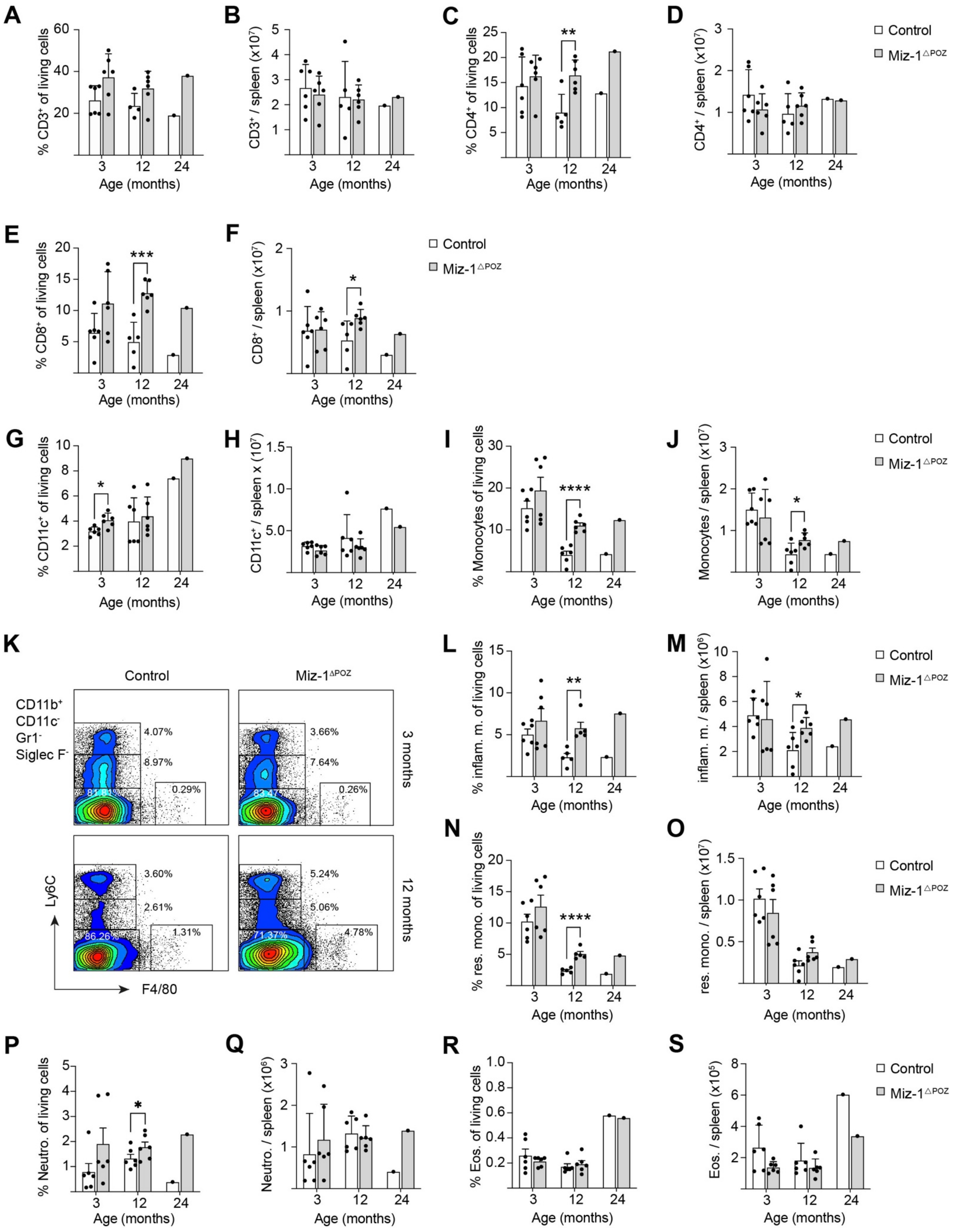

Figure S5. Characterization of splenic lymphocyte populations. (A, C, E) Frequency of splenic $CD3^+$, $CD4^+$ ($CD3^+CD4^+$) and $CD8^+$ ($CD3^+CD8^+$) T cells in the spleen from control and Miz-$1^{\Delta POZ}$ mice. **(B, D, F)** Quantification of splenic $CD3^+$, $CD4^+$ and $CD8^+$ T cells in the spleen from control and Miz-$1^{\Delta POZ}$ mice. **(G and H)** Frequency and quantification of splenic dendritic cells ($CD11c^+$) in control and Miz-$1^{\Delta POZ}$ mice. **(I and J)** Frequency and quantification of splenic monocytes ($CD11b^+CD11c^-Gr\text{-}1^-SiglecF^-Ly6C^+$) in control and Miz-$1^{\Delta POZ}$ mice. **(K)** Flow cytometric analysis of splenocytes from control and Miz-$1^{\Delta POZ}$ mice. Cells were analyzed with antibodies for

$CD11b^+CD11c$, Gr-1, Siglec-F, F4/80 and Ly6C. Percentages in plots are given for the respective gates as percentage of $CD11b^+CD11c^-Gr\text{-}1^-Siglec\text{-}F^-$ cells. (**L and M**) Frequency and quantification of splenic inflammatory monocytes ($CD11b^+CD11c^-Gr\text{-}1^-Siglec\text{-}F^-Ly6C^{high}$) in control and Miz-$1^{\Delta POZ}$ mice. (**N and O**) Frequency and quantification of splenic resident monocytes ($CD11b^+CD11c^-Gr\text{-}1^-Siglec\text{-}F^-Ly6C^{int}$) in control and Miz-$1^{\Delta POZ}$ mice. **(P and Q)** Frequency and quantification of splenic neutrophils ($CD11b^+CD11c^-Gr\text{-}1^+Siglec\text{-}F^-$) in control and Miz-$1^{\Delta POZ}$ mice. **(R and S)** Frequency and quantification of splenic eosinophils ($CD11b^+CD11c^-Gr\text{-}1^-Siglec\text{-}F^+$) in control and Miz-$1^{\Delta POZ}$ mice. Each point represents one mouse. Data are expressed as mean + SD (n≥1). Student's unpaired *t*-test: *p<0.05, **p<0.01, ***p<0.001, ****p<0.0001.

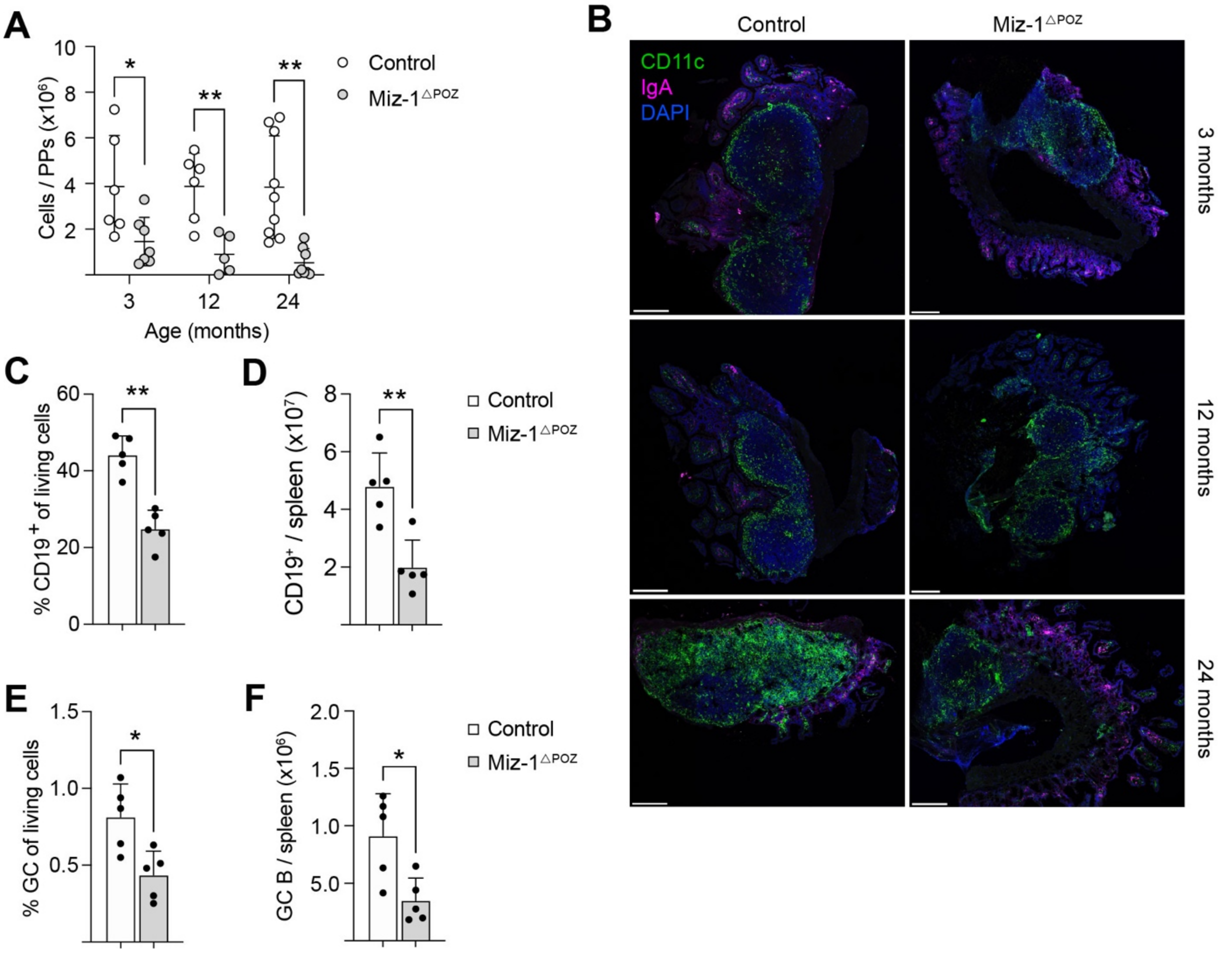

Figure S6. Intestinal immunity and immune response. (A) Cell count of PP from the intestine. **(B)** Immunofluorescence of Peyer's Patches from non-immunized control and Miz-1$^{\Delta POZ}$ at different ages. Cryosections (8 µm) were stained for CD11c (green, follicular dendritic cells), IgA (magenta, antibody secreting cells). DAPI (blue) was used for nuclear counterstain and identification of intestinal epithelial structures lining the luminal surface, characterized by a single layer of linearly arranged nuclei. Scale bar: 200 µm. **(C and D)** Frequency and quantification of splenic CD19^{+} cells in young (3 months) control and Miz-1$^{\Delta POZ}$ mice after boost immunization with NP-CGG. **(E and F)** Frequency and quantification of splenic GC B cells (CD19^{+}GL7^{+}) in young (3 months) control and Miz-1$^{\Delta POZ}$ mice after boost immunization with NP-CGG.
Each point represents one mouse. Data are expressed as mean + SD (n≥2). Student's unpaired t-test: *p<0.05, **p<0.01. For (B) immunofluorescence are representative for 2 independent experiments for each genotype and age.

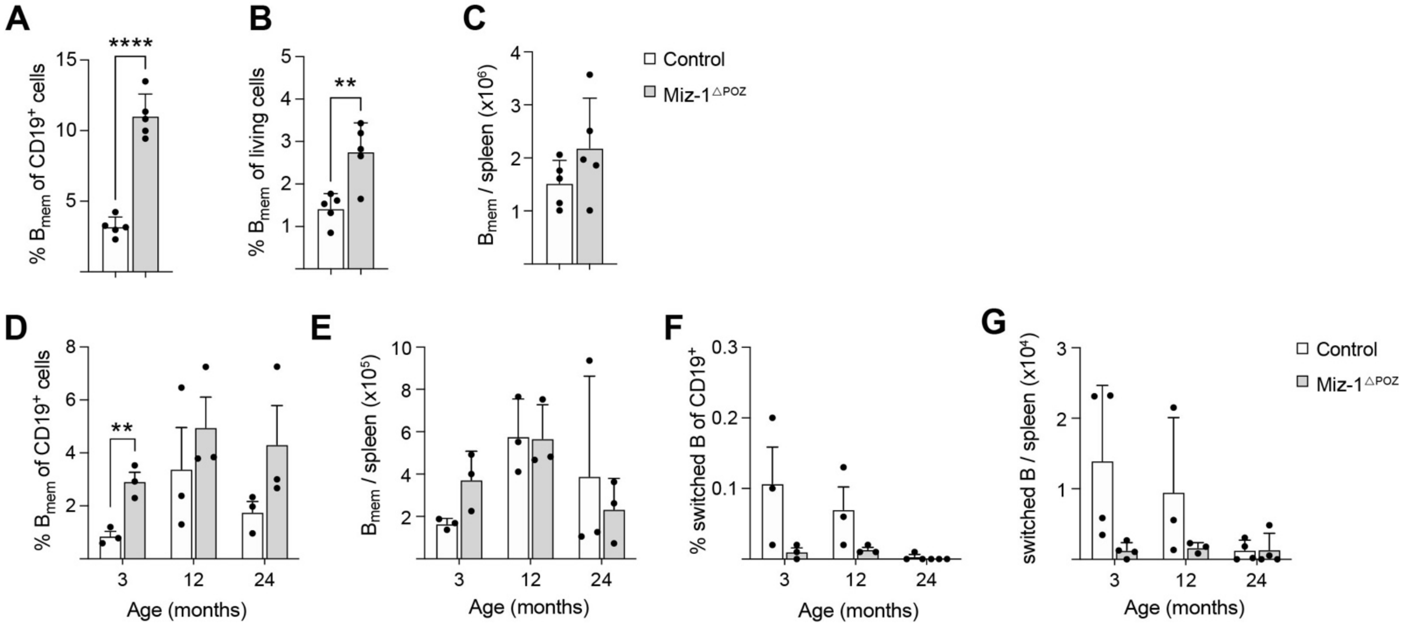

Figure S7. Boosted B_{mem} differentiation. (A-C) Frequency and quantification of splenic B_{mem} cells ($CD19^+GL7^-CD38^+IgG1^+$) in young (3 months) control and Miz-1$^{\Delta POZ}$ mice after boost immunization (d37) with NP-CGG. **(D)** Frequency of memory B cells (B_{mem}, $CD19^+IgD^-IgG1^+CD38^+GL7^-$) in relation to total $CD19^+$ cells in the spleen. **(E)** Quantification of splenic memory B cells (from A) in the spleen from control and Miz-1$^{\Delta POZ}$ mice. **(F)** Frequency of switched B cells ($CD19^+GL7^+CD95^+IgD^-IgG1^+$) in relation to total $CD19^+$ cells in the spleen. **(G)** Quantification of splenic switched B cells (from C) in the spleen from control and Miz-1$^{\Delta POZ}$ mice. Each point represents one mouse. Data are expressed as mean ± SD (n≥3). Student's unpaired t-test: $^{**}p<0.01$, $^{****}p<0.0001$.
